# PRG4CNN: A Probabilistic Model Checking-Driven Robustness Guarantee Framework for CNNs

**DOI:** 10.3390/e27020163

**Published:** 2025-02-03

**Authors:** Yang Liu, Aohui Fang

**Affiliations:** Institute of Logistics Science and Engineering, Shanghai Maritime University, Shanghai 200120, China; lyang@shmtu.edu.cn

**Keywords:** CNN, probabilistic robustness, probabilistic model checking

## Abstract

As an important kind of DNN (deep neural network), CNN (convolutional neural network) has made remarkable progress and been widely used in the vision and decision-making of autonomous robots. Nonetheless, in many scenarios, even a minor perturbation in input for CNNs may lead to serious errors, which means CNNs lack robustness. Formal verification is an effective method to guarantee the robustness of CNNs. Existing works predominantly concentrate on local robustness verification, which requires considerable time and space. Probabilistic robustness quantifies the robustness of CNNs, which is a practical mode of potential measurement. The state-of-the-art of probabilistic robustness verification is a test-driven approach, which is used to manually decide whether a DNN satisfies the probabilistic robustness and does not involve robustness repair. Robustness repair can improve the robustness of CNNs further. To address this issue, we propose a probabilistic model checking-driven robustness guarantee framework for CNNs, i.e., PRG4CNN. This is the first automated and complete framework for guaranteeing the probabilistic robustness of CNNs. It comprises four steps, as follows: (1) modeling a CNN as an MDP (Markov decision processes) by model learning, (2) specifying the probabilistic robustness of the CNN via the PCTL (Probabilistic Computational Tree Logic) formula, (3) verifying the probabilistic robustness with a probabilistic model checker, and (4) probabilistic robustness repair by counterexample-guided sensitivity analysis, if probabilistic robustness does not hold on the CNN. We here conduct experiments on various scales of CNNs trained on the handwriting dataset MNIST, and demonstrate the effectiveness of PRG4CNN.

## 1. Introduction

### 1.1. Motivation

In recent years, DNNs (deep neural networks) have become an effective means to achieve machine learning-based AI (artificial intelligence). As an important kind of DNN, the CNN (convolutional neural network) has made remarkable progress and permeated various facets of autonomous robot operation, including facial recognition [1], autonomous driving [2], natural language processing [3], and video analysis [4]. However, in many scenarios, even a minor perturbation in input for CNN may lead to serious errors, which means CNNs lack robustness. In Goodfellow’s experiments, the original image was classified by GoogLeNet as a panda with 57.7% confidence, and the addition of a small adversarial perturbation to this image resulted in GoogLeNet incorrectly identifying the perturbed image as a gibbon with 99.3% confidence [5]. The problem may also lead to serious consequences in the application of CNNs in some critical settings. For example, in an autonomous driving system, a CNN that incorrectly recognizes a road sign due to a small perturbation may lead the vehicle to make a wrong driving decision, which can lead to serious accidents. In 2016, a crash was caused when a Tesla driverless car incorrectly predicted a white truck as a cloud due to a slightly disturbed image [6]. Despite the demonstrated superior performance and efficiency of CNNs across numerous domains, the robustness of CNNs in practical applications remains a significant concern [7]. At present, there is no uniform or standard definition for the robustness of CNNs. Roughly speaking, it can be divided into three categories: local robustness [8], global robustness [9], and probabilistic robustness [10]. Local robustness indicates that a CNN produces identical predictions for both an original sample and a perturbed sample within a specified local range of perturbations. In practice, the requirement of local robustness is too demanding for CNNs operating in non-malicious environments. Global robustness requires that all inputs in the input space satisfy local robustness. It is not only difficult to control in computation, but is also too demanding for practical use. Probabilistic robustness means that a CNN will accurately predict the perturbed sample within a specified confidence interval, given a range of perturbations for a single sample with an acceptable margin of error. It quantifies the robustness of the CNN, which is a practical potential mode of measurement for determining the robustness of the CNN. Robustness is key to the successful application of a CNN, and non-robust CNNs cannot have widespread application.

Formal verification is a rigorous and complete methodology that mathematically decides whether a system satisfies a certain formal specification (such as, correctness, robustness, safety and reliability). It holds great potential to improve or guarantee the robustness of a CNN. There are some works that try to improve the robustness of CNNs with formal verification. Existing works predominantly concentrate on the formal verification of local robustness for neural networks. The verification process consumes a lot of time and space, due to the non-linear activation functions and complex structure of CNNs. It has been proven to be an NPC problem, even in verifying a simple robust property of a neural network [11]. In 2021, Baluta et al. propose a scalable quantitative robust verification framework for DNNs (deep neural networks). At essence, it is a test-driven approach to provide formal guarantees that a desired probabilistic robustness is satisfied. This is the first practical method for verifying the probabilistic robustness of a DNN. It has the following two main shortcomings: (1) the degree of automation is not high, because it requires much manual testing to prove the soundness of probabilistic robustness; (2) it does not involve robustness repair, but only decides whether a DNN satisfies robustness. To address this limitation, we introduce a novel definition of probabilistic robustness for CNNs, and propose a probabilistic model checking-driven robustness guarantee framework for CNN, i.e., PRG4CNN. It automatically decides whether a CNN satisfies a probabilistic robust property via probabilistic model checking, and repairs the DNN with counterexamples provided by probabilistic model checking if it does not satisfy the conditions. As far as we know, this is the first automated and complete guarantee framework for determining the probabilistic robustness of a CNN.

### 1.2. Related Works

There have been some works on the robustness verification of DNN. We classify them according to what robustness is to be verified. Most existing works focus on local robustness verification. After completing the robustness verification of a DNN, the verification results may show that the DNN does not satisfy robustness. The repair of the DNN can improve the robustness further. We also present related works about the repair of a DNN.

#### 1.2.1. Robustness Verification of DNN

(1) Local Robustness Verification

Bastani et al. employed linear constraints to formulate the robustness verification problem for the entire DNN, effectively transforming it into a satisfiability solving problem [12]. However, Bastani’s approach is limited to a small input region where all ReLU functions are fixed in either activated or inactivated states, thereby only verifying approximations of the desired properties. Narodytska et al. suggest that binarized neural networks can be represented as Boolean formulas and verified robustly using a SAT solver [13]. Nonetheless, this method relies on the premise that SMT verification techniques necessitate the resolution of large-scale constraint problems, which presents significant limitations regarding the extensibility of verification methods that encode robustness verification problems as SMT formulas. Katz et al. introduce Reluplex [14], which extends the simplex method to develop an SMT solver capable of addressing ReLU activation functions, enabling the identification of violations in the nature of counterexamples or the proof of the neural network’s safety. Gehr et al. proposes an extensible ReLU neural network verification framework, AI2 [15], which employs interval abstraction domains and Zonotope abstraction domains to establish a reliable boundary for inputs following perturbation. This framework constructs Zonotope-based abstraction converters to execute various nonlinear operations within the DNN, and ultimately verifies whether the output of the Zonotope meets the specified properties. However, the relaxation of AI2 to the true feasible domain is excessively loose in certain verification scenarios, resulting in an amplification of errors during propagation, and consequently limiting its verification accuracy. Gowal et al. utilized interval boundary propagation to train large neural networks with provable robustness [16]. They introduced an additional axis to the polyhedral constraints for each layer of neurons in the neural network’s parallel upper an approximation bounding box; however, this method is an incomplete algorithm that is applicable solely in training classifiers. Hein et al. proposed the Cross–Lipschitz regularization function [17], which provides robust bounds for neural networks with a single hidden layer by utilizing the local Lipschitz constant. However, for neural networks with multiple hidden layers, this method complicates the derivation of bounds in closed form. Boopathy et al. proposed a general and efficient CNN—Cert framework [18], which extends the robustness verification of deep neural networks to CNNs. It can handle a variety of neural network structures, including convolutional layers, maximum pooling layers, residual blocks, etc., and supports a variety of activation functions. The CNN—Cert framework makes Fast—Lin and CROWN only a special case by taking advantage of the special structure of convolutional layers, and improves their efficiency by 17 and 11 times, respectively, when the robust boundaries are similar or even better. Arcaini et al. introduced a new definition of CNN robustness that addresses unforeseen (but plausible) input modifications [19]. This definition proves easy and effective in evaluating the behavior of CNNs in response to changes in the input image. It seems to be applicable to any field, not just medical image classification. Arcaini et al. also introduced the adversarial definition, which is the vulnerability of binary classifiers to adversarial examples. In addition, Arcaini et al. analyzed different ways to improve the robustness of the network, showing that the best solution is to employ limited data augmentation (LDA) techniques. Ghosh et al. analyzed the performances of some common CNNs for image degradation caused by Gaussian noise, blur, and the use of JPEG and JPEG 2000 compression, covering a range of all quality factors. In addition, Ghosh et al. proposed a master–slave architecture-based approach to improve the performance of CNNs for image classification in the presence of degraded input images [20]. Zhang et al. proposed a general CROWN framework [21] to solve the scalability problem by adding signed linear boundaries to different activation functions in an adaptive relaxation way. CROWN can be extended to neural networks with a variety of activation functions, and even quadratic functions can be used to add appropriate relaxation to activation functions. For ReLU neural networks, CROWN is actually equivalent to the accuracy of DeepPoly. Compared to the previous method, CROWN can effectively validate neural networks with more than 10,000 neurons with different activation functions.

(2) Global Robustness Verification

Ruan et al. defined the global robustness of a DNN as the expected maximum radius of robustness on a test dataset and developed an efficient verification algorithm [9]. Their method involves tensor-based implementation to leverage the intrinsic parallelism of the technique. However, the robust agents computed using this method rely on a limited subset of object operations for recognition. In contrast, Sun et al. introduced DeepGlobal [22], a framework for verifying global robustness in feedforward neural networks (FNNs), which comprises four key components, as follows: a rule generator that identifies all potential boundaries of the network through logical reasoning; a novel network architecture known as the Sliding Door Network (SDN) that facilitates the generation of rules; a mechanism for selecting actual boundaries from the generated potential boundaries; and a filter designed to identify adversarial robust examples (ADRs) through meaningful adversarial instances. The approach proposed by Sun et al. effectively prunes unreachable activation patterns and redundant potential boundaries during the rule generation process, while also utilizing connectivity relations among regions to minimize repetitive operations in the ADR filters, which are steps that incur significant verification costs.

(3) Probabilistic Robustness Verification

PROVERO [23], proposed by Baluta et al., treats the model to be verified as a black box and samples the distribution of the input space to assess the probabilistic robustness of the DNN based on this distribution. PROVERO introduces an extensible framework for the quantitative verification of DNNs, employing a test-driven approach that provides formal guarantees regarding the satisfaction of the desired probabilistic properties. Furthermore, PROVERO presents an algorithm for verifying the quantitative properties of neural networks; however, this algorithm operates under the assumption of black-box access, and the complexity of the test samples is significantly higher than that of comparable baseline algorithms.

#### 1.2.2. Repair of DNN

The repair framework Apricot [24], proposed by Zhang et al., seeks to iteratively repair DNN models through a weight-adaptive approach. The fundamental premise of this framework is that when a deeply learned DNN model is trained on various subsets of the original training dataset, the weights of the resulting simplified deep learning model can yield pertinent information regarding the original model. Although simplified models can mitigate the complexity of the original model, they generally fail to encapsulate the essential features necessary for accurately classifying specific test cases. In contrast, PRDNN [25], introduced by Sotoudeh et al., represents a demonstrable technique for the repair of DNN samples and regions, specifically within polyhedral contexts. Furthermore, PRDNN ensures that the disparity between the original network and the repaired network is minimized during single-layer repairs. However, it is important to note that the sample repair problem, even for single-layer neural networks, is classified as an NPC problem, presenting significant challenges as regards practical solution implementation. Additionally, Sun et al. proposed a causality-based DNN repair technique known as CARE [26]. This technique comprises two primary steps—first, it performs causality-based error localization, and second, it optimizes the parameters of the identified neurons to mitigate the nature of the violations. The causality-based localization strategy focuses on identifying a smaller subset of neurons responsible for the misbehavior that requires repair. This approach differs from gradient-based methods, which rely on statistical correlations and tend to be less interpretable. Following the identification of erroneous neurons, CARE employs a particle swarm optimization algorithm to update the parameters of the identified neurons. This algorithm seeks minor adjustments to the weight parameters of the localized neurons to fulfill the specified properties while preserving the performance of the original network to the greatest extent possible, thereby achieving network repair. Islam et al. utilized a deep neural network program vulnerability dataset, which encompasses 415 neural network program vulnerabilities sourced from Stack Overflow and 555 vulnerabilities from GitHub. They employed this dataset to conduct a comprehensive study on the vulnerability repair patterns of five prominent deep learning libraries, namely Caffe, Keras, TensorFlow, Theano, and PyTorch. In their research, they analyzed several key aspects of neural network program repair models. Firstly, they examined the common vulnerability repair modes, aiming to identify recurring approaches used to address vulnerabilities. Secondly, they investigated the fix patterns associated with multiple vulnerability types, understanding how different types of vulnerabilities are handled in various scenarios. Thirdly, they delve into the remediation mode of vulnerabilities across different deep learning libraries, highlighting any similarities or differences in the way vulnerabilities are addressed within each library. Fourthly, they evaluate the remediation risk, taking into account potential risks that might arise during the remediation process. Lastly, they explore the challenge of fixing neural network program vulnerabilities, shedding light on the difficulties and obstacles faced in rectifying such vulnerabilities [27]. Davide et al. proposed AdRep [28], an adaptive search method for DNN remediation that adaptively updates the target weights during the search process by performing fault location on the current state of the model. They proposed two methods for deciding when to update the target weight, one based on the progress of the fitness value of the search, and the other based on the evolution of the fault location results.

### 1.3. Our Contribution

There are two primary challenges associated with the verification methods for local robustness in CNNs. First, the verified CNNs are predominantly limited to specific network architectures, resulting in computational inefficiencies that demand substantial resources. Second, the outcomes of the verification processes typically yield robustness bounds that the CNN must satisfy or determine whether the CNN maintains robustness within a defined perturbation radius, a criterion that is often excessively stringent.

Global robustness necessitates that all samples within the test set adhere to local robustness criteria, which poses significant computational challenges and renders such approaches impractical. In contrast, the probabilistic approach offers a more lenient definition of robustness. While the verification methods for probabilistic robustness can mitigate some limitations inherent in local robustness verification, they do not provide conclusive evidence that a CNN can sustain its robust characteristics under specific perturbations. To address these issues, there is an urgent need to enhance the verification methods expanding to the robustness of CNNs.

Our work belongs to the probabilistic robustness verification of CNN. Aiming at the shortcomings of state-of-the-art probabilistic robustness verification, we introduce a new definition of probabilistic robustness of CNN, and propose a probabilistic model checking-driven robustness guarantee framework for CNN. The framework can verify the robustness of CNN through probabilistic model checking and repair the CNN by counterexample when the CNN does not meet the corresponding probabilistic robustness. Our method improves the shortcomings of traditional CNN robustness verification methods, such as their low degree of automation and inability to provide a basis for CNN repair. It is the first automated and complete guarantee framework for the probabilistic robustness of CNN, i.e., PRG4CNN. It comprises four primary steps, as follows: (1) construct an MDP (Markov decision processes) model from CNN by model learning, (2) specify the probabilistic robustness of CNN via the PCTL (Probabilistic Computational Tree Logic) formula, (3) verify probabilistic robustness with the probabilistic model checker Prism, (4) repair the CNN by counterexample-guided sensitivity analysis, if probabilistic robustness does not hold on the CNN. It considers the equilibrium between global robustness and probabilistic robustness. In comparison to conventional methods for verifying the robustness of DNNs, our framework PRG4CNN significantly increases the efficiency of verifying CNNs and offers guidance for the robust repair of CNNs.

### 1.4. Structure of the Paper

The subsequent sections of this paper are organized as follows. In Section 2, we present the preliminaries of this work. In Section 3, we propose the PRG4CNN framework in detail and give examples to demonstrate the feasibility. In Section 4, we evaluate our framework PRG4CNN experimentally. Finally, we conclude this paper in Section 5.

## 2. Preliminaries

### 2.1. CNN

The CNN (convolutional neural network) is a feed-forward neural network that contains convolutional operations and has a deep level. This type of network emphasizes the extraction of texture features from images, making it particularly well-suited for applications in computer vision, notably in the domain of image processing. The architecture of a CNN typically comprises a convolutional layer, a pooling layer, and a fully connected layer [29]. Convolutional Layer: In CNNs, the convolutional layer employs convolutional kernels of varying sizes as sensory fields, enabling the network model to scan images and extract features. This process is facilitated through normal distribution initialization, allowing for the learning and updating of convolutional kernel parameters during subsequent backpropagation. Hyperparameters, including kernel size, quantity, stride, and padding, significantly influence the feature extraction capabilities of the CNN. Ultimately, the input image is subjected to a dot product or summation with the weight matrix to generate the feature map for the subsequent processing stage. Pooling Layer: The pooling operation serves as a secondary feature extraction mechanism, functioning similarly to a fuzzy filter. It transforms the original precise descriptions into probabilistic representations, thereby reducing data dimensionality and enhancing model training efficiency. Common pooling operations are categorized into average pooling and maximum pooling. Average pooling involves extracting the mean value of the feature map as the output, whereas maximum pooling selects the maximum value from the feature map as the output. Fully Connected Layer: The fully connected layer, often referred to as the FC layer, is structurally analogous to the hidden layer of a multilayer perceptron. Within the CNN framework, the fully connected layer serves to connect each node in this layer with all nodes in the preceding layer so as to synthesize the features extracted from earlier stages.

### 2.2. Probabilistic Model Checking

Probabilistic model checking is an automatic formal verification technique in the field of system verification. It is specifically designed to decide whether a desired property holds on a probabilistic model. The property is specified via PCTL (Probabilistic Computation Tree Logic) and LTL (Linear Temporal Logic) with probability, and the probabilistic model is DTMC (Discrete-Time Markov Chain), MDP (Markov Decision Process), and so on.

A DTMC [30] is a triple $\left(S,\;I,\;P_{w}\right)$, where S represents a countable memoryless series of states, I represents the initial state distribution, and $P_{w}$ represents the transition probability matrix. If the states in a Markov chain are finite, the number of transfers is finite as well. MDP [31] can be seen as a variant of Markov chains that permits both probabilistic and nondeterministic actions. It can be defined as a quintuple $\left(S,\;I,\;P_{w},\;A\right)$, where S, I and $P_{w}$ denote the same as DTMC, and A denotes the action in MDP. In this work, we assume I has only one initial state.

Computational Tree Logic (CTL) [32] is a prominent branching temporal logic used for specifying system properties. PCTL extends CTL with probability. A PCTL formula formulates conditions on the state of an MDP. The interpretation is Boolean, i.e., a state either satisfies or violates a PCTL formula. PCTL formulae are formed according to the following grammar:

(1) $$\Phi ::=true|a|\Phi_{1}\Lambda \Phi_{2}|\neg \Phi |{Ρ}_{k}(\varphi )$$

(2) φ::=○Φ|Φ1∪Φ2|Φ1∪Φ2≤n

where true denotes a truth value, a denotes an atomic proposition, $\Phi_{1}\Lambda \Phi_{2}$ denotes the union of two formulas, and ¬Φ denotes the negation of a particular formula. ${Ρ}_{k}(\varphi )$ means that the path formula φ holds with some probability in the interval k⊆[0,1]. In path φ,○Φ denotes that the state formula Φ is true in the next state, $\Phi_{1}\cup \Phi_{2}$ indicates that the state formula $\Phi_{1}$ or $\Phi_{2}$ is true in a state of the path, and Φ1∪Φ2≤n indicates that at least one of the state formulas $\Phi_{1}$ or $\Phi_{2}$ is true in the first n state of the path, where n is a natural number.

### 2.3. Adversarial Attacks and Robustness

When introducing the perturbations to input samples, adversarial attacks can lead DNN to produce erroneous predictions regarding these samples. These attacks can be classified into two categories: black-box and white-box attacks [33]. The presence of white-box attacks highlights a significant vulnerability in the robustness of the model. Conversely, black-box attacks are more pertinent to certain practical scenarios. The Fast Gradient Sign Method (FGSM) serves as a classic example of a white-box attack, as shown in Equation (3).

(3) $$x\prime =x+\varepsilon \times sign(\nabla_{x}J(x,y))$$

where x denotes the original sample, x′ denotes the post-perturbation sample, ε denotes the parameter of the perturbation size, sign(⋅) denotes the sign function, $\nabla_{x}J(x,y)$ denotes the objective function, J is the gradient vector with respect to the variable x, and y denotes the true label.

The robustness of a CNN refers to its ability to maintain stable performance and accuracy in the face of various disturbances, noise, or adversarial attacks. We can evaluate the robustness of CNNs in multiple dimensions by means of black-box and white-box attacks. The definition of the probabilistic robustness of a CNN is shown in Inequality (4). It indicates that for a single input sample $x_{0}$ and a perturbed sample with radius r, if the CNN f can make the same recognition result with a probability greater than 1−ε, then the CNN f is said to have ε probabilistic robustness with respect to the perturbation radius r at the sample $x_{0}$.

(4) $$P(\{x\in B_{p}(x_{0},r)|C_{f}(x)=C_{f}(x_{0})\})\geq 1-\varepsilon$$

## 3. Probabilistic Model Checking-Driven Robustness Guarantee

**Figure 1:**
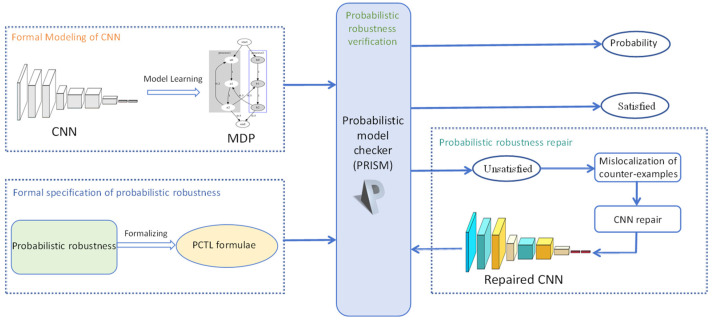
Schematic view of PRG4CNN.

In this section, we propose a probabilistic model checking-driven robustness guarantee framework for CNN, i.e., PRG4CNN, as shown in Figure 1. We exploit probabilistic model checking to decide whether a CNN satisfies the probabilistic robustness, and repair the CNN by counterexample-guided sensitivity analysis if it does not satisfy the probabilistic robustness. This process comprises four components, as follows: formal modeling of CNN by model learning, formal specification of probabilistic robustness as PCTL, probabilistic robustness verification with probabilistic model checker PRISM, and probabilistic robustness repair by counterexample-guided sensitivity analysis. In presenting PRG4CNN, we take a CNN for binary classification tasks as an example, which is noted as Net 0. The network basic structure used for Net 0 is LeNet [34] and the dataset used is the cat and dog classification dataset (https://www.kaggle.com/c/dogs-vs-cats/data, accessed on 20 December 2024) from kaggle. We use 80% of the samples in the dataset as the training dataset and 20% of the samples as the verification dataset. We will consider the probabilistic robustness of this network under the perturbation of three identical attacks with different amplitudes.

### 3.1. Formal Modeling of CNN

We automatically model a CNN as an MDP by model learning. We transform the robustness verification of a CNN to the PCLT probabilistic model checking of an MDP. There are two primary reasons for this. First, MDP, as an abstract model, is generally more interpretable and comprehensible than a CNN itself. Second, an MDP is a frequently used formal model for probabilistic model checking. The established model checking techniques can be employed to decide whether specific properties of the CNN are satisfied, such as security, reachability and robustness.

**Figure 2:**
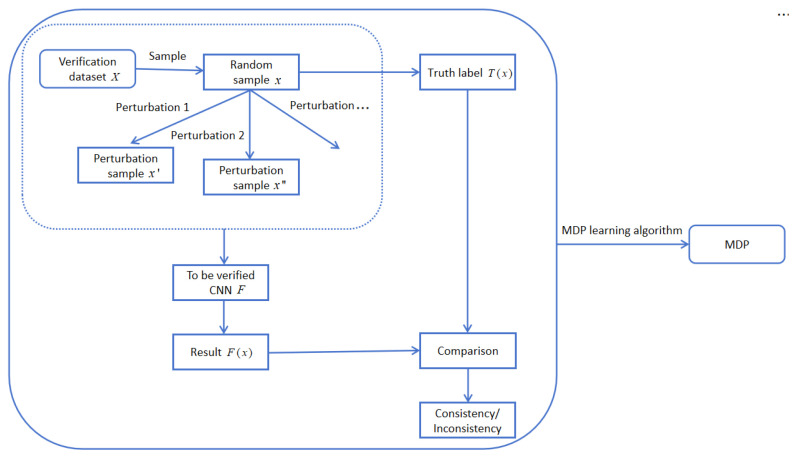
MDP construction process from the CNN.

The learning process of constructing an MDP from a CNN to be verified is shown in Figure 2. X denotes the verification dataset, x denotes a single sample, x′ and x″ denote perturbation samples, F denotes CNN to be verified, F() denotes CNN function, F(x) denotes the prediction result of the CNN for sample x, and T(x) denotes the true label of sample x. The construction of the MDP mainly comprises the following steps: Firstly, the original samples are randomly sampled from the verification dataset, and different types of perturbations are added to the original samples to obtain different types of perturbation samples. Secondly, we compare the prediction results of the original samples and the perturbed samples in the CNN, as well as the truth labels, and construct the MDP through MDP learning algorithm. In order to ensure the validity of the MDP that we learned, it is essential to address three key issues: the selection of states, the selection of actions, and the computation of transition probabilities within the MDP.
**Algorithm 1**: MDP learning algorithm (F, Per, X, N)Input: CNN F to be verified, Perturbation set Per, Verification dataset X,    N Number of samples $\mathrm{Output}:\;\mathrm{MDP}\;(S,\;I,\;P_{w},\;A)$1Determine the MDP action A based on Perturbation set Per2Determine state S based on X and Per3$\mathrm{Init}\;P_{w}$= 0, n= 04Do 5 generate
x in X
6 trace W
7 update P(s,s′) for all8 update 
n
9while
n > 
N
10$\mathrm{Output}\;\mathrm{MDP}(S,\;I,\;P_{w},\;A)$

The states selection of an MDP carries significant implications for modeling CNN. The selected states must encompass the robust properties we intend to analyze within our framework, PRG4CNN. Our objective is to verify the probabilistic robustness of CNN, both in relation to full category samples and category-specific samples. Consequently, the state representation must incorporate perturbed samples, the original samples, and the outcomes produced by the CNN with perturbed samples. Furthermore, it is imperative to include samples from each category, as these state conditions facilitate the analysis of the category-specific robustness.

Other important elements of the learned MDP are the actions. The original sample is added with a perturbation to obtain a perturbed sample, and we use the added perturbation as an action in the MDP.

The transition probability matrix is calculated using repetitive random sampling and monitoring the traces of the input samples. A perturbation is added to the original sample after random sampling, and the CNN predicts the original and perturbed samples. The original samples, the CNN’s prediction results for the original samples, the perturbed samples and the CNN’s prediction results for the perturbed post-samples are taken as a complete trace W. We record all the traces and calculate the transition probability using the frequency. The transition probability matrix $P_{w}$ (estimated based on W) is $P_{w}(s,s\prime )$=$n_{ss\prime }/n_{s}$, where s denotes a state in the MDP, s′ denotes the next state of s, $P_{w}(s,s\prime )$ denotes the transition probability from state s to s′, $n_{s}$ denotes the number of transitions in W originated from state s, and $n_{ss\prime }$ denotes the number of transitions observed from state s to s′ in W. The references [35,36] clarify the number of samples to be used in obtaining a DTMC under probabilistic guaranteed confidence, when the number of samples, N, satisfies Inequality (5), where ε denotes the allowed error, and m denotes the number of states in the MDP, δ′=δ/m. The transition probability of the MDP learned by Algorithm 1 satisfies Inequality (6), where ε denotes the allowed error, δ denotes the confidence level, and $P_{T}(s,s\prime )$ denotes the true transfer probability from state s to s′ in the MDP.

(5) $$N\geq \frac{2}{\varepsilon^{2}}\log(\frac{2}{\delta \prime })[\frac{1}{4}-{\left(\max|\frac{1}{2}-\frac{n_{ss\prime }}{n_{s}}|-\frac{2}{3}\varepsilon \right)}^{2}]$$

(6) $$P(\exists s\in S,|P_{w}(s,s\prime )-P_{T}(s,s\prime )|>\varepsilon )\leq \delta$$

**Figure 3:**
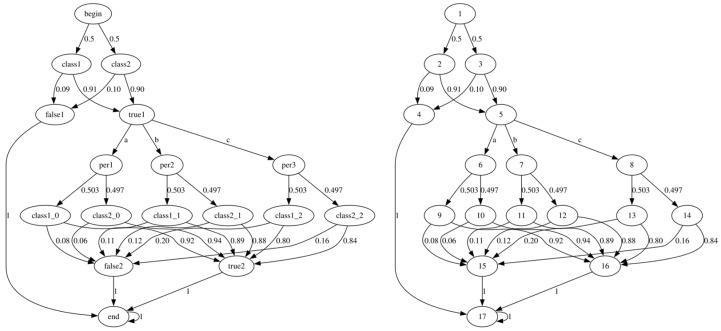
MDP learned from the CNN Net 0 for the binary classification problem.

**Example** **1.** *Let a, b and c denote three different types of perturbations with FGSM perturbation amplitudes of 0.05, 0.075 and 0.1, respectively. To verify the probabilistic robustness of Net 0 under the three perturbations a, b, and c, we randomly divide the verification dataset. The allowable error and confidence level parameters are set as* ε *= 0.05 and* δ *= 0.005, respectively. The number of recorded traces is set as* N *= 30,000. The CNN Net 0 is formally modeled as an MDP using Algorithm 1, which is shown in Figure 3 (left). It contains the start state and the end state constructed in the initialization section, and the individual states of the first category and the second category. The actions of MDP are represented by the added perturbations a, b, and c. The transition probability matrix of the MDP is updated by Algorithm 1 (from line 3 to 9). In order to facilitate the subsequent probabilistic robustness verification in the model checking tool PRISM, we represent the states of MDP using numbers, which are shown in Figure 3 (right).*

### 3.2. Formal Specification of Probabilistic Robustness

The robustness of a CNN refers to its capacity to withstand perturbations and disturbances in the input data. A robust CNN model should be capable of maintaining accurate predictions and demonstrating strong generalization abilities, even in the presence of minor alterations to the input data or noise. At present, there is no universally accepted definition of robustness for CNNs. In this section, we define a new probabilistic robustness for CNN.

**Definition** **1.** *A CNN shows probabilistic robust if the probability of a difference in the correct prediction between the original and perturbed samples is within a given robustness coefficient, under a specific perturbation within the CNN.* *Based on the scope of the verification dataset, we define two types of probabilistic robustness for CNNs, which are also the verified properties. The following are mathematical representations for two types of probabilistic robustness for CNNs.*

Type 1 (Property 1)—probabilistic robustness of CNNs under full category samples,

(7) P(F(x)=T(x)|x⊂X)−P(F(x′)=T(x′)|x′⊂X′)<ξ

Type 2 (Property 2)—probabilistic robustness of CNNs under category-specific samples,

(8) $$P(F(x)=T(x)|x\subset X_{j})-P(F(x\prime )=T(x\prime )|x\prime \subset X_{j}\prime )<\xi$$

It can be seen that Inequality (7) mainly consists of Formulas (9) and (10), and Inequality (8) mainly consists of Formulas (11) and (12). Formula (9) represents the probability that the CNN correctly predicts full category samples. Formula (10) represents the probability that the CNN correctly predicts full category perturbed samples. Formula (11) represents the probability that the CNN correctly predicts category-specific samples. Formula (12) represents the probability that the CNN correctly predicts category-specific perturbed samples.

(9) P(F(x)=T(x)|x⊂X)

(10) P(F(x′)=T(x′)|x′⊂X′)

(11) $$P(F(x)=T(x)|x\subset X_{i})$$

(12) $$P(F(x\prime )=T(x\prime )|x\prime \subset X_{i}\prime )$$

**Theorem** **1.** *Let Quintuple $\left(S,\;I,\;P_{w},\;A\right)$ be MDP M constructed by model learning for a CNN. For any PCTL formula* Φ *, Φ satisfies Inequality (13), where ε denotes the allowed error, δ denotes the confidence level, $P_{T}$ denotes the true transition probability matrix, and $\gamma (P_{w},\;\Phi )$ denotes the probability that $P_{w}$ satisfies the PCTL Φ*.

(13) $$P(|\gamma (P_{w},\Phi )-\gamma (P_{T},\Phi )|>\varepsilon )\leq \delta$$

**Proof** **of** **Theorem** **1.** Triple $\left(S,\;I,\;P_{w}\right)$ in MDP M can be seen as a DTMC. References [30,31] show that a DTMC learned from a DNN satisfies Inequality (13) for any PCTL formula. The MDP M can be viewed as a DTMC with non-determinism. For each deterministic process, the MDP can be viewed as a single DTMC. Therefore, the MDP satisfies Inequality (13) for any PCTL. □

According to the definition of PCTL, it is known that the values of Formulas (9)–(12) can be solved using probabilistic model checking techniques. The probabilistic robustness of the CNN can then be specified by the PCTL formulae, which are obtained from the extension based on Formulas (9)–(12).

**Theorem** **2.** *Let*X *be an estimation of a probability* $X_{t}$ *and satisfy* $P(|X-X_{t}|>\varepsilon )\leq \delta$ *. Let* Z *be an estimation of a probability* $Z_{t}$ *and satisfy* $P(|Z-Z_{t}|>\varepsilon )\leq \delta$*. Then, Inequality (14) holds.*

(14) $$P(|(X-Z)-(X_{t}-Z_{t})|>2\varepsilon )\leq 2\delta -\delta^{2}$$

**Proof** **of** **Theorem** **2.** Knowing $P(|Z-Z_{t}|>\varepsilon )\leq \delta$ and $P(|X-X_{t}|>\varepsilon )\leq \delta$, Inequality (15) and Inequality (16) hold. Simplifying Inequality (15) and Inequality (16) yields a proof. □

(15) $$P(|(X-Z)-(X_{t}-Z_{t})|<2\varepsilon )\geq P(|Z-Z_{t}|<\varepsilon )\cdot P(|X-X_{t}|<\varepsilon )\geq {\left(1-\delta \right)}^{2}$$

(16) $$P(|(X-Z)-(X_{t}-Z_{t})|>2\varepsilon )\leq 1-{\left(1-\delta \right)}^{2}$$

According to Theorem 2, it can be seen that in verifying the robustness of CNNs by PCTL probabilistic model checking, the allowable error and the confidence level of the verification results are changed, and satisfy Inequality (14).

Algorithm 2 is used to generate the PCTL formulae. According to our definition of probabilistic robustness, probabilistic robustness is mainly composed of two-part expressions. We need to determine the starting point and end point, according to the definition of each part of the expression. Finally, based on the two-part PCTL expression and the error coefficient, we obtain a probabilistic robust PCTL formula. Algorithm 2 shows the PCTL formulae generation process. It generates PCTL formulas for the probabilistic robustness representation of the CNN.
**Algorithm 2**: PCTL formulae generationInput: Mathematical representation for probabilistic robustnessOutput: PCTL formulae1Divide the parts of a mathematical representation2Determine start and stop nodes3Get Part of the PCTL4Determination of the parts of the PCTL, robustness coefficient 5Output PCTL

**Example** **2.** *In our running example, taking Net 0 in the above binary classification problem as the CNN to be verified, PCTL formulae of the probabilistic robustness generated via Algorithm 2 are shown in Table 1.*

### 3.3. Probabilistic Robustness Verification

**Figure 4:**
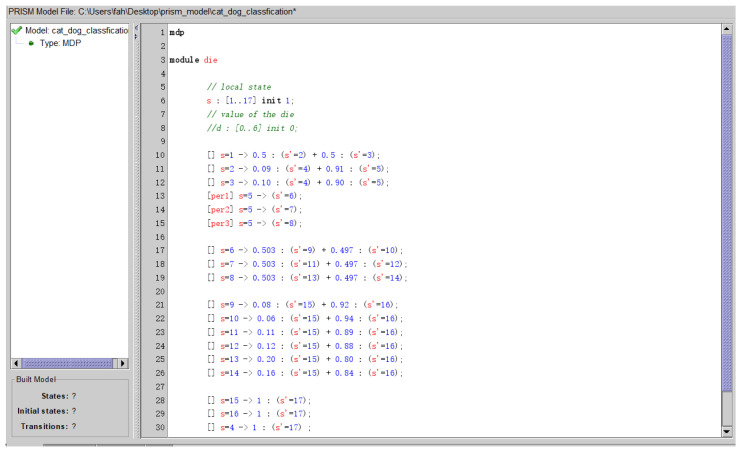
The learned MDP in PRISM.

We transfer the probabilistic robustness verification of CNN to the PCTL probabilistic model checking of MDP. If the MDP satisfies the PCTL formulae of probabilistic robustness, the CNN to be verified satisfies the corresponding probabilistic robustness. If the MDP does not satisfy the PCTL formulae of probabilistic robustness, the CNN to be verified does not satisfy the corresponding probabilistic robustness. As shown in Figure 1, the MDP is learned from the CNN in Section 3.1, PCTL formulae are generated in Section 3.2. We use the famous probabilistic model-checker PRISM to verify the PCTL formulae on MDP. The learned MDP from CNN Net 0 is modeled in PRISM, as shown in Figure 4.

**Figure 5:**
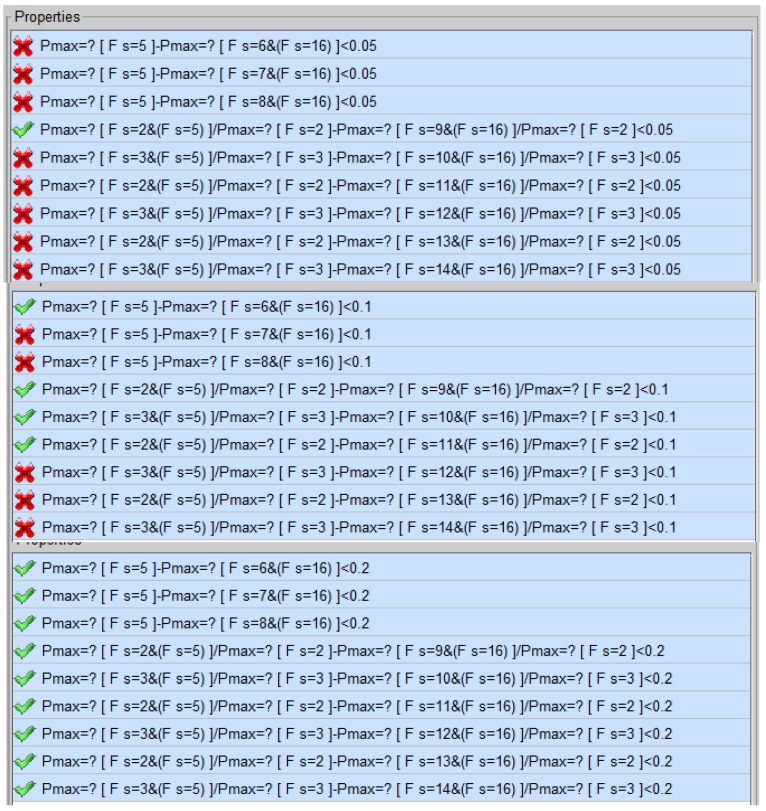
Probabilistic robustness verification results in PRISM.

**Example** **3.** *The verification results are illustrated in Figure 5. When the error coefficient is 0.05, the CNN Net 0 does not satisfy the full category probabilistic robustness of the samples under perturbations of a, b, and c. When the perturbation is a, CNN Net 0 does not satisfy the category-specific probabilistic robustness of category 1. When the error coefficient is 0.1, the CNN Net 0 satisfies the full category probabilistic robustness with perturbation a, and does not satisfy the full category probabilistic robustness under perturbations b and c. With perturbation a, CNN Net 0 does not satisfy category-specific robustness under all categories. With perturbation b, the CNN Net 0 satisfies the category-specific probabilistic robustness of category 1. With a perturbation c, the CNN Net 0 does not satisfy the category-specific probabilistic robustness of category 1 and category 2. When the error coefficient is 0.2, CNN Net 0 satisfies both full category and category-specific probabilistic robustness under perturbations a, b and c, due to the more relaxed conditions. In order to effectively verify the probabilistic robustness of CNN, we need to select an appropriate allowable error coefficient that aligns with the specific context.*

### 3.4. Probabilistic Robustness Repair

**Figure 6:**
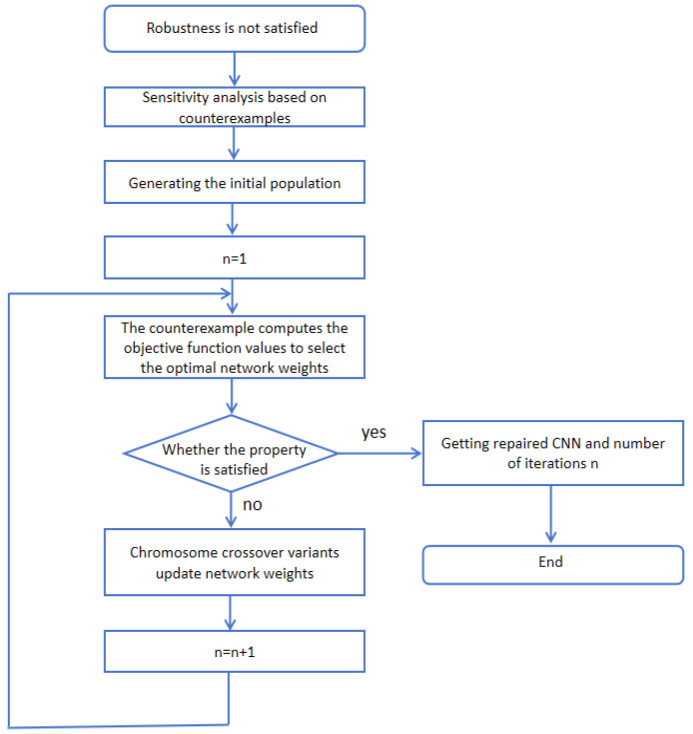
Repair process of CNN.

If the CNN to be verified does not satisfy the probabilistic robustness, we repair the CNN in terms of probabilistic robustness. This is an important component of our framework PRG4CNN. We use counterexample-guided sensitivity analysis to design a Genetic algorithm [37] for iteratively repairing CNN regarding corresponding probabilistic robustness. The repair process is shown in Figure 6, which consists of two primary steps—error localization with counterexamples and CNN weight adjustment by the Genetic algorithm.

The error localization helps to identify the neurons that significantly influence the probabilistic robustness of a CNN. We employ a method grounded in the sensitivity analysis of counterexamples for the error localization of CNNs. When a CNN fails to achieve full category probabilistic robustness, we utilize all samples from the verification set as counterexamples. Conversely, when the CNN does not meet category-specific probabilistic robustness, we restrict the counterexamples to those categories that do not fulfill the probabilistic robustness. Upon identifying a suitable counterexample, we utilize the CNN to generate an aliased prediction of the counterexample, while keeping the weights of specific neurons constant. A lower accuracy rating from this prediction indicates a higher sensitivity of the neuron to the counterexample. This facilitates the identification of the neuron that most significantly affects the probabilistic robustness of the CNN. The sensitivity analyses are presented in Equation (17), where do(x=δ) indicates that the weight of a specific neuron in the CNN is set as δ, y indicates that the CNN is able to classify correctly for the counterexample category samples. The larger value of the formula indicates that the weight of the corresponding neuron has a greater influence on the output result of the CNN.

(17) E[y|do(x=δ)]=∫yp(y|do(x=δ))dy

Repairing CNN is intended to improve its probabilistic robustness. We exploit the Genetic algorithm to adjust the weights of a CNN. Genetic algorithms are a class of evolving algorithms that draw inspiration from the principles of natural evolution. By emulating the processes of natural selection and reproduction, Genetic algorithm can yield high-quality solutions to a diverse array of problems related to search, optimization, and learning. The primary components of a Genetic algorithm encompass coding, the generation of an initial population, fitness evaluation, selection, crossover, and the generation of subsequent populations. We use Equation (18) as the fitness function, where α represents the weight coefficients, Accuracy signifies the accuracy of the CNN on the verification dataset, Probdiff indicates the variance of the probability, which the CNN accurately classifies as a category-specific sample, and $Probdiff_{\max}$ denotes the maximum variance of the probability with which the CNN correctly identifies samples from distinct categories. The lower fitness function indicates superior parameter selection.

(18) $$fitness=\alpha (Probdiff_{\max})+(1-\alpha )(1-Accuracy)$$

**Example** **4.** *In our running example, the findings from Example 3 indicate that the category-specific probabilistic robustness for category 1 is not achieved by the CNN with perturbation b, which has a robustness coefficient of 0.1. Furthermore, when perturbation type c is applied, the category-specific probabilistic robustness of the CNN fails to meet both category 1 and category 2. Consequently, category 1 is utilized as the counterexample for perturbation b, while both category 1 and category 2 serve as counterexamples for perturbation c. The CNN is subsequently repaired using Figure 6, and the repair results are presented in Table 2. The data in the table demonstrate that our method significantly enhances the classification robustness of the CNN across various perturbations.*

## 4. Experiments and Analysis

### 4.1. Experiment Setup

We conduct our experiments with PyCharm 2022.1.3 and PRISM 4.8.1. The version of the python compiler we are using is python 3.6. The computer is a multi-core Intel Core i7 4.7 GHz CPU with 16 GB memory. The verified CNN models are four CNNs that have been trained on the MNIST dataset [38]. The MNIST dataset comprises a set of handwritten digit images commonly used in machine learning. It contains 60,000 training images and 10,000 test images, all of which are 28 × 28 pixel grayscale images covering handwritten 0–9. In MNIST, each image corresponds to a label from 0 to 9 indicating the number written. In this experiment, the 10 handwritten data categories in the MNIST dataset correspond to categories 1 to 10, respectively. Table 3 shows the specific information about the CNNs to be verified.

We take LeNet [34] as Net 1. LeNet is a classical CNN architecture proposed by Yann LeCun et al. It is one of the early deep learning models used for handwritten digit recognition, and played an important role in advancing the development of computer vision. LeNet consists of two main parts—a convolutional layer and a fully connected layer.

We take DenseNet [39] as Net 2. DenseNet is a densely connected CNN proposed by Gao Huang et al. Unlike traditional CNNs, each layer in DenseNet is connected to all previous layers, which imparts the network with a stronger feature reuse capability and gradient mobility. DenseNet refers to the output of each layer as a “dense block”, and inside the dense block, the input of each layer comes not only from the previous layers but also from all previous layers. In this way, the outputs of all layers are passed directly to the subsequent layers, avoiding information loss and redundant computation.

We take VGG11 [40] as Net 3. VGG11 is a model based on a deep CNN architecture, proposed by Karen Simonyan and Andrew Zisserman. VGG11 is one of the VGGNet family, which has a simpler structure but still achieves good performance.

We take ResNet18 [41] as Net 4. ResNet18 is a classical Residual Network (RN) architecture proposed by Kaiming He et al. It is one of the representative models of deep residual learning, which solves the problems of gradient vanishing and gradient explosion in deep neural network training by introducing residual connections.

### 4.2. Verification Results and Analysis

Utilizing the PRG4CNN method as proposed in Section 3, the CNN that has been previously trained serves as the subject of verification, while the test set from the MNIST dataset is employed as the verification set. We verify the probabilistic robustness of the four CNNs in the context of adversarial attacks with FGSM as the attack strategy. The perturbation amplitudes selected for FGSM are 0.025, 0.05, 0.075 and 0.1, respectively. The allowable error ε and the confidence level δ are set as 0.01 and 0.001, respectively. Additionally, the number of recorded traces, denoted as N, is established at 40,000. The allowable error coefficient ξ is set as 0.1. The results of the verification are presented in Table 4.

Table 4 indicates a sequential increase in the number of trainable parameters across the networks Net 1, Net 2, Net 3, and Net 4, which corresponds to an increase in network complexity. Furthermore, the verification results of four CNNs in response to the FGSM attack demonstrate that more complex CNNs exhibit superior probabilistic robustness, compared to the simpler CNNs, although they have largely equivalent training parameters.

### 4.3. CNN Repair

As shown in Section 4.2, when the robustness coefficient is set to 0.1, none of the four CNNs achieve category-specific probabilistic robustness in the presence of perturbations characterized by an FGSM amplitude 0.1. To address the counterexample associated with the CNN, we employ the repair method shown in Section 3.4. The outcomes of the repaired CNN are shown in Table 5. The data in the table demonstrate that our framework PRG4CNN significantly enhances the probabilistic robustness of CNNs as regards the specified categories. Furthermore, even in scenarios where the accuracy of CNN remains relatively low following perturbation, the framework PRG4CNN markedly improves the probabilistic robustness of CNNs for all categories.

## 5. Conclusions

In this paper, we propose a framework PRG4CNN to guarantee the probabilistic robustness of CNNs. This framework comprises four key components: the formal modeling of CNNs, the formal specification of probabilistic robustness, probabilistic robustness verification and probabilistic robustness repair. This addresses certain limitations of traditional methods for verifying CNNs, broadens the verification techniques available for assessing CNN robustness, and offers insights into the repair of CNN for probabilistic robustness. We demonstrate the effectiveness of our framework PRG4CNN via various CNNs with the handwriting dataset MNIST. It is important to note that the learned MDP primarily reflects the input and output of a CNN, but does not present the detailed internal structure of the CNN. According to our definition of probabilistic robustness, we choose different perturbations to construct the MDP. How to choose the appropriate perturbation is a challenging problem that we need to study further. In addition, the current application of our method is limited to the classification of CNNs. In the future, we will develop a learning algorithm to create a more accurate model for CNN. Moreover, we will extend our framework PRG4CNN to guarantee the robustness of other CNNs, and to guarantee the probabilistic robustness of other DNNs.

## Figures and Tables

**Table 1:** PCTL formulae of probabilistic robustness for CNN Net0.

Property	Meaning	PCTL Formulae
Property 1	Probabilistic robustness under perturbation a	Pmax = ? [F s = 5]−Pmax=? [F s = 6&(F s = 16)] < ξ
	Probabilistic robustness under perturbation b	Pmax = ? [F s = 5 ]−Pmax=? [F s = 7&(F s = 16)] < ξ
	Probabilistic robustness under perturbation c	Pmax = ? [F s = 5]−Pmax = ? [F s = 8&(F s = 16)] < ξ
Property 2	Probabilistic robustness of sample category 1 under perturbation a	Pmax = ? [F s = 2&(F s = 5)]/Pmax = ? [F s = 2]−Pmax = ? [F s = 9&(F s = 16) ]/Pmax = ? [F s = 2] < ξ
	Probabilistic robustness of sample category 2 under perturbation a	Pmax = ? [F s = 3&(F s = 5)]/Pmax = ? [F s = 3]−Pmax = ? [F s = 10&(F s = 16)]/Pmax = ? [F s = 3] < ξ
	Probabilistic robustness of sample category 1 under perturbation b	Pmax = ? [F s = 2&(F s = 5)]/Pmax = ? [F s = 2]−Pmax = ? [F s = 11&(F s = 16)]/Pmax = ? [F s = 2] < ξ
	Probabilistic robustness of sample category 2 under perturbation b	Pmax = ? [F s = 3&(F s = 5)]/Pmax = ? [F s = 3]−Pmax = ? [F s = 12&(F s = 16)]/Pmax = ? [F s = 3] < ξ
	Probabilistic robustness of sample category 1 under perturbation c	Pmax = ? [F s = 2&(F s = 5)]/Pmax = ? [F s = 2]−Pmax = ? [F s = 13&(F s = 16)]/Pmax = ? [F s = 2] < ξ
	Probabilistic robustness of sample category 2 under perturbation c	Pmax = ? [F s = 3&(F s = 5)]/Pmax = ? [F s = 3]−Pmax = ? F s = 14&(F s = 16)]/Pmax = ? [F s = 3] < ξ

**Table 2:** CNN verification results after the repair.

CNN Name	Perturbation	Robustness Coefficient	Property 1	Accuracy	Property 2	Probdiff_max_
Net 0	b	0.1	satisfied	0.752->0.813	satisfied	0.124->0.089
Net 0	c	0.1	unsatisfied	0.7390->0.786	unsatisfied	0.194->0.117

**Table 3:** CNNs to be verified.

CNN Name	CNN Type	Dataset	Model Size	Number of Parameters	Training Epoch
Net 1	Lenet	MNIST	82 k	21,840	10
Net 2	Densenet	MNIST	4.18 M	1,000,618	10
Net 3	Vgg11	MNIST	35.2 M	9,231,114	10
Net 4	Resnet18	MNIST	42.7 M	11,173,962	10

**Table 4:** Verification results of four CNNs.

CNN Name	Perturbation Type	Amplitude	Robustness Coefficient	Property 1	Property 2	Property 2 Counterexample
Net 1	FGSM	0.025	0.1	satisfied	satisfied	
Net 1	FGSM	0.05	0.1	satisfied	unsatisfied	category 7, 9
Net 1	FGSM	0.075	0.1	unsatisfied	unsatisfied	category 3–10
Net 1	FGSM	0.1	0.1	unsatisfied	unsatisfied	category 1–10
Net 2	FGSM	0.025	0.1	satisfied	satisfied	
Net 2	FGSM	0.05	0.1	satisfied	satisfied	
Net 2	FGSM	0.075	0.1	satisfied	satisfied	
Net 2	FGSM	0.1	0.1	satisfied	unsatisfied	category 3
Net 3	FGSM	0.025	0.1	satisfied	satisfied	
Net 3	FGSM	0.05	0.1	satisfied	satisfied	
Net 3	FGSM	0.075	0.1	satisfied	satisfied	
Net 3	FGSM	0.1	0.1	satisfied	unsatisfied	category 10
Net 4	FGSM	0.025	0.1	satisfied	satisfied	
Net 4	FGSM	0.05	0.1	satisfied	satisfied	
Net 4	FGSM	0.075	0.1	satisfied	satisfied	
Net 4	FGSM	0.1	0.1	satisfied	unsatisfied	category 3

**Table 5:** Verification results of four CNNs after the repair.

CNN Name	Perturbation Type	Amplitude	Property 1	Accuracy	Property 2	Probdiff_max_
Net 1	FGSM	0.05	satisfied	0.9427->0.9384	satisfied	0.113->0.076
Net 1	FGSM	0.1	satisfied	0.8516->0.9023	unsatisfied	0.175->0.112
Net 2	FGSM	0.05	satisfied	0.9541->0.9527	satisfied	0.071->0.051
Net 2	FGSM	0.1	satisfied	0.8826->0.9245	satisfied	0.167->0.092
Net 3	FGSM	0.05	satisfied	0.9527->0.9512	satisfied	0.082->0.053
Net 3	FGSM	0.1	satisfied	0.8729->0.9351	satisfied	0.152->0.072
Net 4	FGSM	0.05	satisfied	0.9648->0.9615	satisfied	0.082->0.047
Net 4	FGSM	0.1	satisfied	0.9219->0.9514	satisfied	0.137->0.056

## Data Availability

The data presented in this study are available on request from the corresponding author.

## Nomenclature

**Symbol Definition**	**Meaning**
X	Verification dataset
x	A sample in verification dataset
F()	CNN function representation
F(x)	CNN prediction results for sample x
T(x)	True labeling of sample x
$X_{i}$	The true category of samples is category i
δ	Perturbation
X′	Perturbation sample set, X′:X′=X+δ
x′	A sample after perturbation
N	Number of samples
ξ	Robustness coefficient

## References

[1] Bhatta A., Mery D., Wu H., Annan J., King M.C., Bowyer K.W. Our deep CNN face matchers have developed achromatopsia. Proceedings of the IEEE/CVF Conference on Computer Vision and Pattern Recognition.

[2] Lv Z., Qiao L., Yang S. (2022). Memory-augmented neural networks based dynamic complex image segmentation in digital twins for self-driving vehicle. Pattern Recognit..

[3] Das S., Imtiaz M., Neom N.H. (2023). A hybrid approach for Bangla sign language recognition using deep transfer learning model with random forest classifier. Expert Syst. Appl..

[4] Zhou W., Liu M., Xu Z. (2022). The dual-fuzzy convolutional neural network to deal with handwritten image recognition. IEEE Trans. Fuzzy Syst..

[5] Goodfellow I.J., Shlens J. (2015). Explaining and harnessing adversarial examples. Stat.

[6] Golson J. (2016). Tesla driver killed in crash with Autopilot active. Verge. https://www.theverge.com/2016/6/30/12072408/tesla-autopilot-car-crash-death-autonomous-model-s.

[7] Li L., Xie T., Li B. (2023). Certified robustness for deep neural networks. IEEE Symp. Secur. Priv. (SP).

[8] Zhong Z., Tian Y., Ray B. Understanding local robustness of deep neural networks under natural variations. Proceedings of the Fundamental Approaches to Software Engineering: 24th International Conference.

[9] Ruan W., Wu M., Sun Y. Global robustness evaluation of deep neural networks with provable guarantees for the hamming distance. Proceedings of the IJCAI-19.

[10] Mangal R., Nori A.V., Orso A. Robustness of neural networks: A probabilistic and practical approach. Proceedings of the 2019 IEEE/ACM 41st International Conference on Software Engineering: New Ideas and Emerging Results (ICSE-NIER).

[11] Kaveh M., Mesgari M. (2023). Application of meta-heuristic algorithms for training neural networks and deep learning architectures: A comprehensive review. Neural Process. Lett..

[12] Bastani O., Ioannou Y. (2016). Measuring neural net robustness with constraints. Adv. Neural Inf. Process. Syst..

[13] Narodytska N., Kasiviswanathan S., Ryzhyk L. Verifying properties of binarized deep neural networks. Proceedings of the AAAI Conference on Artificial Intelligence.

[14] Katz G., Barrett C., Dill D.L. Reluplex: An efficient SMT solver for verifying deep neural networks. Proceedings of the Computer Aided Verification: 29th International Conference, CAV 2017.

[15] Gehr T., Mirman M., Drachsler-Cohen D. Ai2: Safety and robustness certification of neural networks with abstract interpretation. Proceedings of the 2018 IEEE Symposium on Security and Privacy (SP).

[16] Gowal S., Dvijotham K.D., Stanforth R. Scalable verified training for provably robust image classification. Proceedings of the IEEE/CVF International Conference on Computer Vision.

[17] Hein M., Ndriushchenko M. (2017). Formal guarantees on the robustness of a classifier against adversarial manipulation. Adv. Neural Inf. Process. Syst..

[18] Boopathy A., Weng T.W., Chen P.Y., Liu S., Daniel L. Cnn-cert: An efficient framework for certifying robustness of convolutional neural networks. Proceedings of the AAAI Conference on Artificial Intelligence.

[19] Arcaini P., Bombarda A., Bonfanti S., Gargantini A. Dealing with robustness of convolutional neural networks for image classification. Proceedings of the 2020 IEEE International Conference on Artificial Intelligence Testing (AITest).

[20] Ghosh S., Shet R., Amon P., Hutter A., Kaup A. Robustness of Deep Convolutional Neural Networks for Image Degradations. Proceedings of the 2018 IEEE International Conference on Acoustics, Speech and Signal Processing (ICASSP).

[21] Zhang H., Weng T.W., Chen P.Y., Hsieh C.J., Daniel L., Efficient Neural Network Robustness Certification with General Activation Functions NuerIPS 2018. https://github.com/IBM/CROWN-Robustness-Certification?tab=readme-ov-file.

[22] Sun W., Lu Y., Zhang X. (2022). DeepGlobal: A framework for global robustness verification of feedforward neural networks. J. Syst. Archit..

[23] Baluta T., Chua Z.L., Meel K.S. Scalable quantitative verification for deep neural networks. Proceedings of the 2021 IEEE/ACM 43rd International Conference on Software Engineering (ICSE).

[24] Zhang H., Chan W.K. Apricot: A weight-adaptation approach to fixing deep learning models. Proceedings of the 2019 34th IEEE/ACM International Conference on Automated Software Engineering (ASE).

[25] Sotoudeh M., Thakur A.V. Provable repair of deep neural networks. Proceedings of the 42nd ACM SIGPLAN International Conference on Programming Language Design and Implementation.

[26] Sun B., Sun J., Pham L.H., Shi J. Causality-based neural network repair. Proceedings of the 44th International Conference on Software Engineering.

[27] Islam M.J., Pan R., Nguyen G., Rajan H. Repairing deep neural networks: Fix patterns and challenges. Proceedings of the ACM/IEEE 42nd International Conference on Software Engineering.

[28] Li Calsi D., Duran M., Laurent T., Zhang X.Y., Arcaini P., Ishikawa F. (2023). Adaptive Search-based Repair of Deep Neural Networks. Proceedings of the Genetic and Evolutionary Computation Conference (GECCO ’23).

[29] Li Z., Liu F., Yang W. (2021). A survey of convolutional neural networks: Analysis, applications, and prospects. IEEE Trans. Neural Netw. Learn. Syst..

[30] Jones G., Qin Q. (2022). Markov chain Monte Carlo in practice. Annu. Rev. Stat. Its Appl..

[31] Kurniawati H. (2022). Partially observable markov decision processes and robotics. Annu. Rev. Control Robot. Auton. Syst..

[32] Clarke F.M., Henzinger T.A., Veith H. (2018). Handbook of Model Checking.

[33] Wang Y., Liu J., Chang X., Wang J., Rodríguez R.J. (2022). AB-FGSM: AdaBelief optimizer and FGSM-based approach to generate adversarial examples. J. Inf. Secur. Appl..

[34] Al-Jawfi R. (2009). Handwriting Arabic character recognition LeNet using neural network. Int. Arab J. Inf. Technol.

[35] Bazille H., Genest B., Jégourel C. Global PAC bounds for learning discrete time Markov chains. Proceedings of the Computer Aided Verification: 32nd International Conference, CAV 2020.

[36] Sun B., Sun J., Dai T. Probabilistic verification of neural networks against group fairness. Proceedings of the Formal Methods: 24th International Symposium, FM 2021.

[37] Sohail A. (2023). Genetic algorithms in the fields of artificial intelligence and data sciences. Ann. Data Sci..

[38] Deng L. (2012). The mnist database of handwritten digit images for machine learning research: A guideline and a benchmark study. IEEE Signal Process. Mag..

[39] Zhang K., Guo Y., Wang X. (2019). Multiple feature reweight densenet for image classification. IEEE Access.

[40] Sengupta A., Ye Y., Wang R. (2019). Going deeper in spiking neural networks: VGG and residual architectures. Front. Neurosci..

[41] He K., Zhang X., Ren S. Deep residual learning for image recognition. Proceedings of the IEEE conference on computer vision and pattern recognition.

